# High precision multi-genome scale reannotation of enzyme function by EFICAz

**DOI:** 10.1186/1471-2164-7-315

**Published:** 2006-12-13

**Authors:** Adrian K Arakaki, Weidong Tian, Jeffrey Skolnick

**Affiliations:** 1 Center for the Study of Systems Biology, School of Biology, Georgia Institute of Technology, Atlanta, Georgia 30318, USA; 2 Department of Biological Chemistry and Molecular Pharmacology, Harvard Medical School, Boston, Massachusett 02115, USA

## Abstract

**Background:**

The functional annotation of most genes in newly sequenced genomes is inferred from similarity to previously characterized sequences, an annotation strategy that often leads to erroneous assignments. We have performed a reannotation of 245 genomes using an updated version of EFICAz, a highly precise method for enzyme function prediction.

**Results:**

Based on our three-field EC number predictions, we have obtained lower-bound estimates for the average enzyme content in Archaea (29%), Bacteria (30%) and Eukarya (18%). Most annotations added in KEGG from 2005 to 2006 agree with EFICAz predictions made in 2005. The coverage of EFICAz predictions is significantly higher than that of KEGG, especially for eukaryotes. Thousands of our novel predictions correspond to hypothetical proteins. We have identified a subset of 64 hypothetical proteins with low sequence identity to EFICAz training enzymes, whose biochemical functions have been recently characterized and find that in 96% (84%) of the cases we correctly identified their three-field (four-field) EC numbers. For two of the 64 hypothetical proteins: PA1167 from *Pseudomonas aeruginosa*, an alginate lyase (EC 4.2.2.3) and Rv1700 of *Mycobacterium tuberculosis *H37Rv, an ADP-ribose diphosphatase (EC 3.6.1.13), we have detected annotation lag of more than two years in databases. Two examples are presented where EFICAz predictions act as hypothesis generators for understanding the functional roles of hypothetical proteins: FLJ11151, a human protein overexpressed in cancer that EFICAz identifies as an endopolyphosphatase (EC 3.6.1.10), and MW0119, a protein of *Staphylococcus aureus *strain MW2 that we propose as candidate virulence factor based on its EFICAz predicted activity, sphingomyelin phosphodiesterase (EC 3.1.4.12).

**Conclusion:**

Our results suggest that we have generated enzyme function annotations of high precision and recall. These predictions can be mined and correlated with other information sources to generate biologically significant hypotheses and can be useful for comparative genome analysis and automated metabolic pathway reconstruction.

## Background

Genome sequencing, gene identification and the functional annotation of gene products are the basic first steps towards understanding the wide spectrum of biological processes taking place in a living organism. Although each of these steps presents its own difficulties, the experimental determination of protein function is probably the most challenging [1]. It is practically impossible to perform functional assays for all the uncharacterized proteins provided by the hundreds of genome sequencing projects that are currently underway. Computational tools are clearly necessary to assist in a task of such great magnitude [2]. In fact, the functional descriptions associated with the vast majority of genes in newly released genomes are not experimentally determined but are simply inferred from similarity to previously characterized sequences [3-5]. The basic assumption of this strategy, often (and misleadingly) referred to as "annotation transfer by homology" [6], is that sequence similarity implies functional similarity. However, the transfer of function based on sequence similarity is complicated by a technical issue: the lack of consistent annotation strategies, and by two other main factors: functional divergence and the domain organization of proteins.

Functional divergence of highly similar sequences has been detected in many protein families [7,8]. In these cases, the use of permissive criteria to assess the significance of the similarity between proteins can easily lead to wrong annotations. For example, detailed biochemical function is not completely conserved between similar proteins even at 60% [9] to 70% pairwise sequence identity [10]; however, much lower thresholds have been used in the functional annotation of some genomes [11]. On the other hand, the modularity of proteins and the fact that different domains of the same protein may have different functions [12] can also lead to wrong functional assignments, for example, when the domain structure of a best database hit is overlooked [13]. These two factors associated with functional annotation based on sequence similarity represent the most important sources of error in genome annotation [13-15]. The consequences of these misannotations are far reaching because they propagate in public databases [15], leading to their systematic deterioration, a process termed error percolation [16]. Genome reannotation, i.e. the annotation of a previously annotated genome using better bioinformatics algorithms and more complete databases [3], provides more accurate and up-to-date functional information and can mitigate the effects of error percolation when the higher quality annotations reach the databases [16]. Genome reannotation projects can provide improved gene structure, refinement of function annotation, benchmarking or comparison of different annotation strategies, and evaluation of annotation reproducibility [3]. In this spirit, we focus here on the reannotation of biochemical function as a more quantifiable aspect of this general problem.

The biological function of a protein can be defined in a physiological, developmental, cellular or biochemical context, among others [5]. From a biochemical point of view, the most important group of proteins is constituted by enzymes. Enzymes are responsible for the majority of biochemical functions, catalyzing the chemical reactions involved in the metabolism of all living organisms and represent a significant fraction of a proteome [17]. Enzymes are organized according to the Enzyme Commission (EC) system, a hierarchical classification that assigns unique four-field numbers to different enzymatic activities [18]. The first field of an EC number indicates the general class of catalyzed reaction: 1. oxidoreductases, 2. transferases, 3. hydrolases, 4. lyases, 5. isomerases and 6. ligases. The second and third fields depend on different criteria related to the chemical features of the substrate and the product of the reaction, and the fourth field is a sequential number without any special meaning. An EC number is assigned solely based on the global reaction that is catalyzed and does not provide information about a specific catalytic mechanism, evolutionary family or structural fold associated to the classified enzyme [19]. New schemes that overcome these problems of the EC system are under development [20,21]; however, their impact on the biological community is very low compared to the widespread recognition and the universal use of the EC classification. For example, all the main databases focused on enzymes (e.g. BRENDA [22] and ENZYME [23]) or metabolic pathways (e.g. KEGG, the Kyoto Encyclopedia of Genes and Genomes [24] and MetaCyc [25]) rely on the EC classification.

In our previous work [26], we presented EFICAz (Enzyme Function Inference by a Combined Approach), an engine for large-scale enzyme function inference that addresses the limitations of annotation approaches based on sequence similarity alone. EFICAz combines different methods based on family-dependent sequence similarity thresholds, the presence of patterns in functionally relevant domains, and the identification of functionally discriminating residues, all carefully optimized to generate highly precise predictions (see Methods and our previous article [26] for a detailed description of EFICAz). In this work, we present the results of a multi-genome scale reannotation of enzyme function, using an updated version of EFICAz.

Many genome reannotation efforts have been dedicated to individual species [3,27-30]; here, we investigate 245 genomes, in a very consistent way, and obtain EC number annotations for more than 200,000 coding sequences predicted to be enzymes by EFICAz; more than 14,000 of these are novel functional predictions.

Freilich and collaborators have recently conducted a survey and analysis of enzymes in 85 genomes [17]; however, they have inferred enzyme function using strategies based on sequence similarity alone, which suffer from the problems mentioned above. On the contrary, in this study, we employ EFICAz, a method that we specifically designed and optimized to generate high quality predictions [26]. The ultimate purpose of our multi-genome reannotation project is to provide detailed enzyme function assignments, i.e. four-field EC numbers when possible or at least three-field EC numbers, that permit the reconstruction of metabolic pathways. Accordingly, we have generated a detailed, precise and standardized biochemical function annotation of genome sequences that satisfy the strong requirements of automated methods for metabolic pathway reconstruction [31]. In fact, based on the results of the present reannotation study, we have initiated our own multi-genome scale metabolic pathway reconstruction project, where we demonstrate that novel EFICAz assignments permit the connection of a significant number of disjointed subpathways that occur systematically in certain groups of bacterial species (manuscript in preparation). However, we believe that the up-to-date enzyme function annotations obtained from this large-scale analysis, all available on our website [32], will also be of great utility to researchers interested in comparative genome analysis or the general understanding of biochemical processes occurring in particular species.

This manuscript is organized as follows: in the Results and Discussion section, we first present a reassessment of enzymatic content in organisms from the three domains of life. Second, we compare our predictions with enzyme function annotations from two releases of KEGG. Third, we estimate the precision of our novel assignments by comparing EFICAz predicted and experimentally derived biochemical functions of 64 previously hypothetical proteins. Fourth, we provide examples that highlight the potential of novel EFICAz predictions as a source of biologically relevant hypotheses. In the Conclusions section, we summarize the present work, stress its significance, and discuss its limitations. Finally, in the Methods section, we briefly describe EFICAz, introduce the data sources of our analysis, present the results of an extensive benchmark in a scenario of periodic updates, and describe the procedure we followed to identify recently characterized hypothetical proteins.

## Results and discussion

### Enzyme content assessed by EFICAz

We applied our enzyme function prediction method (EFICAz version 5.0) to the 245 genomes available in the *Genes *database Release 33.0+/03–05 of KEGG [24]. In Table 1, we show taxonomic information, scientific names and KEGG genomes abbreviations for all the species analyzed in this study, which include representatives from the three domains of life [33]: 21 archaeal species, 204 bacterial species and 20 eukaryotic species. EFICAz assigned four-field (three-field) EC numbers to 158,941 (221,999) of the 866,142 coding sequences found in the set of analyzed genomes. All the EFICAz predictions are available on our website [32], where they can be downloaded and browsed by various criteria (see next section). The multi-genome scale application of EFICAz not only provides a highly precise annotation of enzyme function, it also gives us the opportunity of reassessing the enzyme content throughout the different domains of life in a very consistent way.

**Figure 1:**
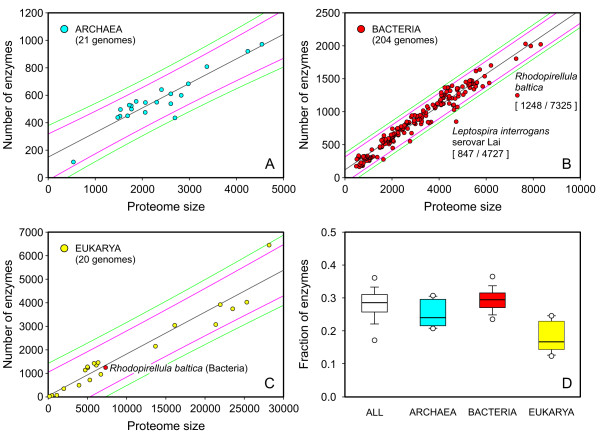
**Enzyme content in organisms from the three domains of life**. Number of enzymes as a function of the proteome size for archaeal (**A**), bacterial (**B**) and eukaryotic (**C**) genomes. The gray, magenta and green lines represent: regression line, 95% and 99% prediction intervals, respectively. (**D**) Distribution of the fraction of enzymes in archaeal, bacterial and eukaryotic genomes. The statistics represented in the box-and-whisker plots are: outliers below the 10th percentile (circles, bottom), 10th percentile (whisker, bottom), 25th percentile (box, bottom), median (thick line), 75th percentile (box, top), 90th percentile (whisker, top) and outliers above 90th percentile (circles, top).

In Figure 1, we show the number of enzymes per genome (estimated by the number of sequences annotated with three-field EC numbers by EFICAz) as a function of proteome size, for archaeal (Fig. 1A), bacterial (Fig. 1B) and eukaryotic (Fig. 1C) species. As reported before based on analyses of a smaller set of genomes [17,34], we observe a linear relationship between the number of enzymes and proteome size that is characteristic of genomes in each domain of life; although we note that those of archaeal and eukaryotic species are very similar (correlation coefficient R^2 ^= 0.85, 0.95 and 0.93, regression coefficient or slope *b *= 0.179, 0.242 and 0.178, standard error of regression coefficient *se*_*b *_= 0.017, 0.004 and 0.012, intercept a = 149.24, 118.56 and 42.04 for archaeal, bacterial and eukaryotic genomes, respectively). Only two bacterial organisms, *Rhodopirellula baltica *and *Leptospira interrogans *serovar Lai, show a significant deviation from the linear relationship, both having less enzymes than expected for their proteome size (Fig. 1B). The main feature shared by these two species is an elevated number of regulatory proteins as a consequence of adaptation to changing environments. *Rhodopirellula baltica*, the only planctomycetes among the analyzed genomes, has acquired a high proportion of two-component systems and Extra Cytoplasmic Function sigma factors to adapt to the changing conditions of free-living in marine, fresh water and terrestrial environments [35,36]. Interestingly, the enzyme content of *Rhodopirellula baltica *agrees very well with the linear relationship between the number of enzymes and eukaryotic proteome size (Fig. 1C), yet another eukaryotic-like feature of planctomycetes in addition to the lack of peptidoglycan in their cell walls, unique cell compartmentalization, and presence of a condensed fibrillar nucleoid [35].

The spirochaete *Leptospira interrogans *serovar Lai, a pathogenic non-obligate parasitic bacterium that can survive as a saprophyte or as a facultative parasite of mammals, has developed a vast regulatory system to interpret the signals from these distinct environment [37,38]. The other serotype of *Leptospira interrogans *analyzed in our set, serovar Copenhageni [39], would also show less enzymes than expected from a linear relationship if a minimum open reading frame (ORF) size less restrictive than 50 amino acids would have been used for ORF detection (less than 4% of the analyzed genomes show a minimum ORF size so high). The low enzyme content of *Rhodopirellula baltica *and *Leptospira interrogans *compared with other bacterial organisms of similar proteome size is thus consistent with the correlation that has been reported between the low fraction of enzymes and the massive recruitment of regulatory proteins [17,40]. Figure 1D shows the distribution of the fraction of enzymes characteristic of each domain of life, whose median and mean ± standard deviation values are: 0.24 and 0.25 ± 0.04 for Archaea, 0.29 and 0.30 ± 0.05 for Bacteria, and 0.17 and 0.18 ± 0.05 for Eukarya. Freilich and collaborators have recently reported higher estimates for the fraction of enzymes in the three domains of life [17]; however, the sets they analyzed included enzymes and some non-enzymes, because their definition of putative enzyme was much more permissive than the one used in our analysis. Thus, we can consider theirs and ours as upper-bound and lower-bound estimates of the true values, respectively. See additional file 1: Enzyme_content.xls for a list of the estimated fraction of enzymes for each of the analyzed genomes.

### Comparison of EFICAz predictions with KEGG annotations

**Figure 2:**
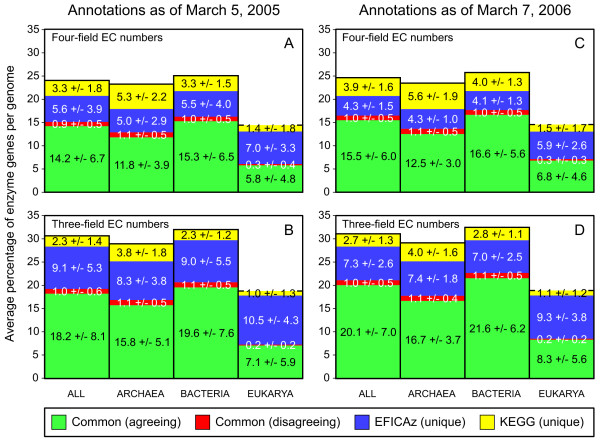
**Comparison of EFICAz predictions with KEGG annotations**. Comparison of EFICAz predictions with KEGG annotations from the *Genes *database of March 5, 2005, Release 33.0+/03–5 (**A-B**) and of March 7, 2006, Release 37.0+/03–07 (**C-D**). We analyze two levels of enzyme function description: four-field EC numbers (**A, C**) and three-field EC numbers **(B, D)**. For all, archaeal, bacterial and eukaryotic genomes we plot the average percentage of enzymatic proteins per genome whose EFICAz-inferred and KEGG-provided annotations at the specified level of detail agree (green columns) or disagree (red columns), and whose enzyme function annotation at the specified level of detail is only provided by EFICAz (blue columns) or by KEGG (yellow columns). The numeric values inserted in each stacked column are the corresponding average percentage of enzymatic proteins per genome +/- the standard deviation.

To evaluate the level of agreement of EFICAz predictions with other sources of annotation, we compared our enzyme function assignments to those available in the *Genes *database of KEGG. In general, the quality and completeness of the functional annotation of genomes tend to continuously improve due to the incessant flow of new experimental results and the correction of systematic errors in annotation transfer [13]. To account for the dynamic nature of the functional annotation process, we compare our predictions with annotations from two different releases of the *Genes *database: (i) 33.0+/03–05 of March 5, 2005, which is contemporary to the sources we employed for training the version of EFICAz used for our multi-genome scale enzyme annotation effort (Fig. 2A, B), and (ii) 37.0+/03–07, released a year later (Fig. 2C, D). We compare the enzyme function annotations at the level of four-field EC numbers (Fig. 2A, C) and three-field EC numbers (Fig. 2B, D), in the latter case, we compare only the first three fields of the annotated EC numbers, whether the fourth field is known or unknown. Besides our EFICAz predictions, we also have the set of KEGG annotations as of 2006 available on our website [32], where the assignments made by EFICAz and/or KEGG can be browsed. The annotations can also be easily selected and retrieved according to species name, level of detail of the enzyme function prediction (four-field or three-field EC numbers), consistency or inconsistency between EFICAz and KEGG assignments, presence of the keywords "hypothetical" or "unknown" in KEGG assignments as of 2005, EC number and gene name.

The functional annotations in the *Genes *database of KEGG is obtained from various sources: descriptions of gene functions in the GenBank [41] database, on-line genome databases which are generally more up-to-date, the Swiss-Prot [42] database, and additional annotations by KEGG based on ortholog identification and pathway reconstruction [43]. Since the algorithms for enzyme function annotation employed by EFICAz and KEGG are different, and their sources only partially overlap, it is expected to find some sequences for which both methods make functional assignments (which may agree or disagree), and other sequences for which only one of the methods is capable of making an EC number assignment. In Figure 2, we plot the average percentage of sequences per genome for which EFICAz enzyme function predictions and KEGG annotations agree (green) or disagree (red) at the four- or three-field EC number level, and the average percentage of sequences per genome for which only EFICAz (blue) or KEGG (yellow) provide enzyme function information at the specified level of detail. The statistical significance of the differences observed after one year in the mean percentage of annotations corresponding to each group of EC number assignments was evaluated by correlated two-tailed t-tests at a critical alpha level of 10^-3^.

#### Most newly added KEGG enzyme function annotations agree with earlier EFICAz predictions

We first analyze the degree of agreement of the enzyme function assignments for sequences that both EFICAz and KEGG annotate as enzymes. As of 2005, we observe that, on average, EFICAz and KEGG assign the same four- and three-field EC numbers to 14.2% (Fig. 2A) and 18.2% (Fig. 2B) of the sequences in a proteome, respectively. Only an average of 0.9% (Fig. 2A) and 1.0% (Fig. 2B) of the sequences in a proteome show disagreement in their four-field and three-field EC number assignments, respectively.

When KEGG annotations as of 2006 are considered, the agreement increases from 14.2% (Fig. 2A) to 15.5% for four-field EC number assignments (Fig. 2C) and from 18.2% (Fig. 2B) to 20.1% for three-field EC number assignments (Fig. 2D). In contrast, there is no significant change in the level of disagreement. After one year, the average percentage of sequences in a proteome with four-field (three-field) EC numbers assigned by KEGG, including agreeing, disagreeing and unique annotations, grew from 18.4% (21.6%) to 20.3% (23.8%), with 67% (80%) of that growth corresponding to agreeing annotations and only 2% (1%) corresponding to disagreeing annotations. Thus, most of the newly added enzyme function annotations in KEGG agree with predictions made by EFICAz a year before. About 31% (19%) of the growth corresponds to unique four-field (three-field) EC number annotations made by KEGG, which are analyzed in the next section.

#### EFICAz predictions have higher coverage than KEGG annotations, especially for eukaryotes

Figure 2 shows that the average fraction of sequences per proteome that is only annotated by KEGG with four-field (three-field) EC numbers increased from 3.3% to 3.9% (2.3% to 2.7%) after one year. Still, unique EFICAz predictions are more numerous than unique KEGG annotations, even when KEGG annotations as of 2006 are considered. The average fraction of sequences per proteome with three-field (four-field) EC number assignments made only by EFICAz is 4.0 (1.7) times higher than the fraction corresponding to KEGG as of 2005, and 2.7 (1.1) times higher than the fraction corresponding to KEGG as of 2006.

When we analyze the unique predictions in genomes from different domains of life, the most extreme difference between the number of EFICAz-based and KEGG-based unique assignments corresponds to Eukarya. For eukaryotic genomes, the average fraction of unique EFICAz predictions ranges from 4.0 (Fig. 2C) to 10.9 (Fig. 2B) times higher than the average fraction of unique KEGG annotations. Before suggesting an explanation for this discrepancy, we should mention that one of the principles used by KEGG curators for enzyme function annotation is the transfer of annotation between orthologs, which are identified by sequence similarity with consideration of the positional coupling of genes on the chromosome [43]. Thus, a probable reason for the low number of KEGG-based unique assignments in eukaryotes is that in these organisms, with only a few exceptions [44], genes do not appear to be organized in operons, preventing KEGG annotators from making use of the conservation of local genomic context (such as gene order or gene neighboring) to validate orthology-based annotations of enzyme function. This observation raises the question as to how much the recall of EFICAz would improve if we account for the conservation of local genomic context. We would expect an increased coverage for archaeal and bacterial genomes; although, evidently, this component of the method would not be relevant for enzyme function inference of single sequences.

### EFICAz predictions for recently characterized hypothetical proteins

**Figure 3:**
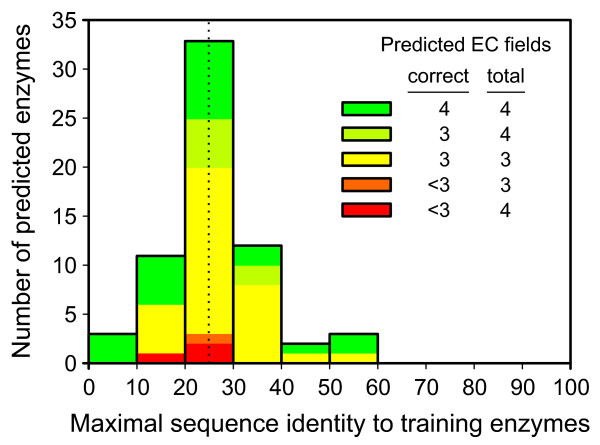
**Similarity of 64 previously hypothetical proteins to EFICAz training enzymes**. Number of previously hypothetical proteins predicted to be enzymes by EFICAz at different intervals of maximal sequence identity to enzymes included in the EFICAz version 5.0 training set. The true enzyme function of these 64 previously hypothetical proteins has been recently determined; therefore, we could assess the precision of our predictions. Dark green, light green and red bars represent four field EC number predictions with four, three or less than three correct EC fields, respectively. Yellow and orange bars represent three field EC number predictions with three or less than three correct EC fields, respectively. The median of the distribution (24.8%) is indicated by the broken line.

In the previous section, we have shown that a considerable fraction of an average proteome is annotated with at least three-field EC numbers only by EFICAz. An average of 36%, 25% and 12% of the three-field EC number annotations uniquely provided by EFICAz in the archaeal, bacterial and eukaryotic proteomes, respectively, correspond to proteins annotated as hypothetical in KEGG as of 2005. In this section, we assess the EFICAz predictions for a subset of hypothetical proteins for which experimentally-derived enzyme function annotation has recently become available. More precisely, we compare the EFICAz-predicted and the experimentally-derived EC numbers of 64 proteins annotated as hypothetical in KEGG whose enzyme functions we could confidently retrieve from the literature (see Methods for details). For this evaluation, we assume that the true EC number associated to an enzyme is the one derived from the referred experimental results. To exclude cases in which the transfer of functional annotation could be successfully achieved in most cases by simple sequence similarity based methods, we only consider hypothetical proteins whose maximal sequence identity to any of the enzymes we used to train EFICAz is less than 60%. We have previously shown that below this threshold of sequence identity the conservation of enzyme function is on average poor [9]. From the histogram shown in Figure 3, we can observe that the median value of the maximal sequence identity to training enzymes is only 25%.

#### EFICAz correctly predicts the enzyme function of most of the recently characterized hypothetical proteins

EFICAz could predict four-field EC numbers for 37 of the 64 previously hypothetical proteins analyzed. We further divided these 37 proteins in two groups: one group of 25 proteins for which the number of matching first fields between the EFICAz-predicted and the true EC numbers can be univocally determined (Table 2), and another group of 12 proteins for which the number of matching fields could be either three or four (Table 3). We observe that the four fields of the predicted and the true EC numbers agree for 21 of the 25 proteins listed in Table 2, indicating a precision of 84% for EFICAz four-field EC number prediction applied to this set of hypothetical proteins. The three-field precision of EFICAz four-field EC number predictions is 92%, since 34 out of 37 proteins listed in Tables 2 and 3 show agreement in at least the first three fields of the predicted and true EC numbers. Table 4 lists 27 of the 64 previously hypothetical proteins analyzed, for which EFICAz could only predict three-field EC numbers. In this case, 26 out of 27 proteins show agreement in the first three fields of the predicted and the true EC numbers, indicating a precision of 96% for EFICAz three-field EC number prediction applied to these hypothetical proteins.

In agreement with the results of the benchmark described in Methods, there is no significant correlation between the precision of the EFICAz predictions for this set of hypothetical proteins and their sequence similarity to the enzymes included in the EFICAz training set (Figure 3). Also, the precision agrees reasonably well with the average precision derived from the benchmark test, especially considering the small size of the analyzed sample (64 proteins), and the fact that hypothetical proteins that are the subject of recent publications often belong to novel families. For example, three of our five wrong predictions correspond to enzymes that are the first studied member of a new family, with no significant sequence similarity to other functionally equivalent proteins: (i) the product of gene MJ0044 of *Methanococcus jannaschii *(Table 2), an isopentenyl-phosphate kinase that still has not been assigned an EC number by the Enzyme Commission [18], (ii) the product of gene MJ0936 of *Methanococcus jannaschii *(Table 2), a new cAMP phosphodiesterase, and (iii) the product of Ta1419 gene of *Thermoplasma acidophilum*, a novel bifunctional phosphoglucose/phosphomannose isomerase (Table 3). It is well known that Archaea have unique enzymes that are optimized for extreme environments [45]; therefore, it is not surprising that these three misclassified proteins belong to archaeal organisms.

#### The annotation lag in databases can be longer than two years

Interestingly, for some of the 64 previously hypothetical proteins analyzed, the experimental evidence to support a specific enzyme function has been available for quite a long time in the literature; however, the corresponding functional annotation is not acknowledged in current databases. One of the 21 successfully predicted enzymes listed in Table 2, the product of the PA1167 gene from *Pseudomonas aeruginosa*, constitutes an example of this problem, known as annotation lag [46]. An article available as early as May 10, 2004 describes the biochemical characterization of PA1167 and demonstrates that it is a new alginate lyase (EC 4.2.2.3), an alginate biofilm degrading enzyme [47]. However, as of June 27, 2006, PA1167 was still annotated as a hypothetical protein in all the relevant databases we checked, from very general ones such as Swiss-Prot (Accession number: Q9I4H0) [42] and Entrez Gene (GeneID: 878215) [48], to those that are genome-oriented such as KEGG (Entry: PA1167 of *Pseudomonas aeruginosa*) [24] and TIGR-CMR, The Institute for Genomic Research Comprehensive Microbial Resource (TIGR Locus: NT03PA1297) [49], and even a database exclusively dedicated to *Pseudomonas aeruginosa*, Pseudomonas Genome Database v2 (Locus ID: PA1167) [50]. We think this specific example is worth mentioning, given the direct involvement of alginate biofilm in the pathogenicity of this bacterial species, and the recently suggested therapeutic possibilities of alginate lyase in the treatment of *Pseudomonas aeruginosa *infection of respiratory tract in cystic fibrosis patients [51]. Similarly, experimental evidence supporting the ADP-ribose diphosphatase activity (EC 3.6.1.13) of the product of gene Rv1700 of *Mycobacterium tuberculosis *H37Rv (Table 2) has been available since August, 2003 [52,53]; however, it is currently annotated as a hypothetical protein in all major databases. We believe that more elaborate approaches for detecting these ignored but highly confident functional assignments (e.g. methods based on natural-language processing of full text journal articles [54]) would extract considerably more annotations than our simple keyword-based PubMed search (see Methods for details).

### Utility of novel predictions made by EFICAz

The results of the thorough benchmark described in Methods, the agreement between newly added enzyme function annotations in KEGG and EFICAz predictions made a year earlier, and the precision of EFICAz predictions for recently characterized hypothetical proteins, suggest that novel predictions made by EFICAz are of high confidence and can provide interesting leads for investigation in many biological fields. Below, we present two interesting cases that exemplify the utility of EFICAz predictions for hypothetical proteins. We believe that experts in different fields of biology will be capable of formulating other interesting hypothesis based on the mining of our numerous novel predictions.

#### EFICAz predictions as hypothesis generators for understanding functional roles of hypothetical proteins

Although not biochemically characterized even in the most recent literature, some of the hypothetical proteins that EFICAz predicts to be enzymes are known to be directly or indirectly involved in specific biological processes. In these cases, the enzyme function predicted by EFICAz can help to form new hypotheses about the functional role of a hypothetical protein in the particular biological process with which it has been associated. To illustrate this situation, we selected the product of the human gene FLJ11151. As of June 27, 2006, FLJ11151 was annotated as a hypothetical protein in Swiss-Prot (Accession number: Q9BRF8) [42], Entrez Gene (GeneID: 55313) [41] and KEGG (Entry: 55313 of *Homo sapiens *genome) [24], and lacked any kind of functional description in the Ensembl v39 database (Vega Gene ID: OTTHUMG00000073008) [55].

The enzyme function of FLJ11151 predicted by EFICAz is endopolyphosphatase (EC 3.6.1.10). Endopolyphosphatases catalyze the non-processive internal cleavage of polyphosphate (chain of tens to hundreds of phosphate residues linked by phosphoanhydride bonds [56]) to release polyphosphate chains of shorter size [57]. Endopolyphosphatase activity has been detected in all eukaryotes tested to date, from unicellular organisms like *Saccharomyces cerevisiae *to mammals [57]; however, no human gene has been shown to be associated to this enzymatic activity or proposed as a putative endopolyphosphatase before this work. Although it was recently shown that the terminal cleavage products of the *Saccharomyces cerevisiae *endopolyphosphatase Ppn1 are inorganic phosphate and triphosphate, the K_m _value of Ppn1 for polyphosphate chains of 45 to 20 phosphate residues is much higher than its K_m _for long chains [58]. Therefore, under physiological conditions, Ppn1 probably degrades the long-chain polymer to short-chain polyphosphate of more than 20 phosphate residues, which is known to be required for the growth of yeast in minimal medium [59].

The physiological role of short-chain polyphosphate in mammals is unclear; however, *in vitro *experiments have demonstrated that polyphosphate chains of 15 to 750 residues strongly activate the serine/threonine kinase mTOR (mammalian Target Of Rapamycin) [60]. Activation of mTOR kinase, a central regulator that integrates growth factor and nutrient signals, enhances tumor growth and neoplastic proliferation [61]. Consequently, its inhibition is a cancer therapeutic strategy that is being vigorously investigated [62]. *In vivo *experiments have shown that the activation of mTOR by polyphosphate can be suppressed in human carcinoma cell lines by the expression of a highly processive exopolyphosphatase of yeast (EC 3.6.1.11) that degrades the polymer to inorganic phosphate [63], resulting in a dramatic reduction of cell proliferation [60].

Interestingly, the FLJ11151 transcript has been found to be expressed at high tag count in four Serial Analysis of Gene Expression (SAGE) libraries of primary melanomas in the vertical or metastatic growth phase, indicating that the hypothetical protein FLJ11151 may play an important role in advanced stages of cancer [64]. The predicted endopolyphosphatase activity of FLJ11151 suggests that the product of this gene may be involved in tumorigenesis via an activation of mTOR. We propose that the activation is due to an increased level of short-chain polyphosphate produced by the cleavage of longer molecules of the polymer. It is important to emphasize that we have arrived at this hypothesis in a semi-automatic way, by correlating the results of a PubMed [65] search for a given gene with its EFICAz-predicted enzyme function.

#### Candidate virulence factors predicted by EFICAz

Most of the virulence factors detected in pathogenic organisms exhibit some kind of enzymatic activity, e.g. many exotoxins are pentosyltransferases (EC 2.4.2.-), serine endopeptidases (EC 3.4.21.-) or metalloendopeptidases (EC 3.4.24.-) [66]. Furthermore, some carboxylic ester hydrolases (EC 3.1.1.-) and phosphoric diester hydrolases (EC 3.1.4.-) are involved in invasion or host cell penetration [67], and several peptidases (EC 3.4.-.-) are implicated in anti-immune strategies to evade the host defenses [68]. Even some housekeeping enzymes that perform essential metabolic functions can also play a role in enhancing virulence in many pathogens [69]. Thus, hypothetical proteins whose EFICAz-predicted enzyme functions are known to be associated with pathogenicity can be considered as putative virulence factors. The product of the gene MW0119 of *Staphylococcus aureus *strain MW2 and its ortholog SA0140 in strain N315, both annotated as sphingomyelin phosphodiesterases by EFICAz, constitute a good example of this type of novel prediction. N315 and MW2 are meticillin resistant *S. aureus *(MRSA) strains, that were isolated from hospital-acquired [70] and community-acquired infections [71], respectively. The treatment of patients infected by MRSA has become increasingly difficult because MRSA strains are beginning to develop resistance to vancomycin, the antibiotic traditionally used to treat MRSA infections [72].

*S. aureus *is the human pathogen that displays the widest assortment of virulence factors [73]. Beta-hemolysin, beta-toxin or sphingomyelinase C, one of the many exotoxins secreted by *S. aureus*, is a sphingomyelin phosphodiesterase (EC 3.1.4.12) that disrupts the membranes of erythrocytes and other mammalian cells [74]. In humans, beta- hemolysin has been shown to selectively kill monocytes, which then release cytokines that are important for the initiation and progression of *S. aureus *infection [75]. As of June 27, 2006, only truncated beta-hemolysins were annotated in the *S. aureus *MW2 genome: the product of the genes MW1881 (TIGR Locus: NT03SA2038, Swiss-Prot accession number: Q99QS0, Entrez GeneID: 1003995, KEGG entry: MW1881 of *S. aureus *MW2 genome) and MW1940 (TIGR Locus: NT03SA2101, Swiss-Prot accession number: Q8NVM0, Entrez GeneID: 1004054, KEGG entry: MW1940 of *S. aureus *MW2 genome). Similarly, the only beta-hemolysins annotated in the genome of strain N315 were truncated: the product of the genes SA1752 and SA1811, orthologs of MW1881 and MW1940, respectively. The inactivation of the beta-hemolysin genes in MW2 and N315 strains is caused by the insertion of bacteriophages [76].

The apparent absence of active beta-hemolysins in the MW2 and N315 strains opens the possibility that other genes with sphingomyelinase activity could serve as their functional substitutes. MW0119, one of our predicted sphingomyelin phosphodiesterases, was annotated as a "hypothetical protein, similar to lactococcal phosphatase homologue" in TIGR-CMR (TIGR Locus: NT03SA0129) [49], lacked a functional annotation in Swiss-Prot (Accession number: Q8NYQ6) [42] and Entrez Gene (GeneID: 1004871) [41], and was annotated as a hypothetical protein in KEGG (Entry: MW0119 of *S. aureus *MW2 genome) [24]. In all these databases, the annotations for SA0140, the ortholog of MW0119 in strain N315, were identical to those of MW0119. Based on the enzymatic activity assigned by EFICAz to these gene products, we suggest that the hypothetical proteins MW0119 and SA0140 may act as beta-hemolysins in the MW2 and N315 strains of *S. aureus*. We believe that the EFICAz-based strategy of detecting putative virulence factors described here can generate leads for the developing of new antibacterial agents, which are urgently needed given the increasing magnitude of the public health problem that multiresistance to antibiotics constitute.

## Conclusion

The reannotation effort presented in this work provides up-to-date enzyme function information corresponding to 245 genomes. Based on the fact that more than double the number of genomes considered in previous analyses are now available [17,34], and using EFICAz, our highly precise approach for enzyme function prediction, we have confirmed the existence of a linear relationship between the number of enzymes and proteome size and provided up to date estimations of the fraction of enzymes in genomes from each domain of life (Figure 1).

**Figure 4:**
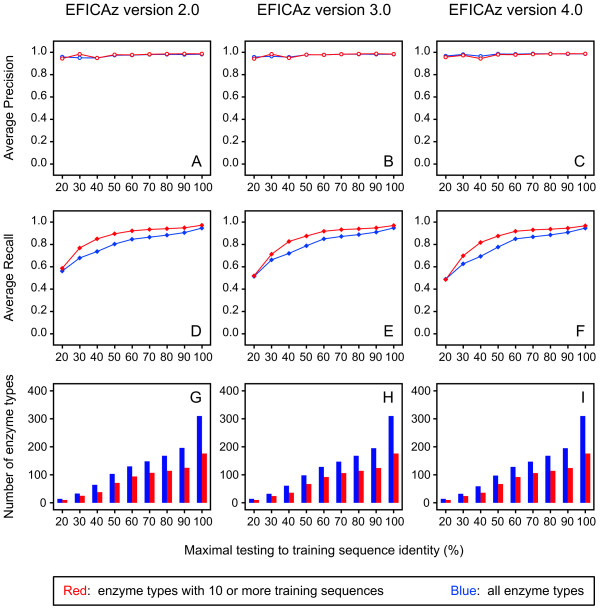
**Benchmark test of updated versions of EFICAz**. Precision (**A-C**), recall (**D-F**) and number of enzyme types described by four-field EC numbers (**G-I**) for different versions of EFICAz, at different levels of maximal testing to training sequence identity, averaged per enzyme type. Curves in red correspond to enzyme types for which at least 10 training sequences were available; curves in blue correspond to all enzyme types. The training of versions 2.0, 3.0 and 4.0 of EFICAz is based on the Releases 2.0, 3.0 and 4.0 of UniProt, respectively. The new Swiss-Prot sequences added to UniProt 5.0 since the release of UniProt 2.0, 3.0 and 4.0 constitute the test sequences for versions 2.0, 3.0 and 4.0 of EFICAz. See Methods for a full description of the benchmark procedure.

Precision was the highest priority of our analysis; accordingly, our results suggest that by using EFICAz [26], we have generated annotations of good quality. First, the comprehensive series of benchmarks of EFICAz show that we can expect a mean precision of 94% regardless of the sequence similarity between testing and training enzymes (Figure 4A–C). Second, by comparing our predictions with KEGG annotations available a year later (which can take advantage of updated databases and new experimental results available in the literature), we find that most of the newly added KEGG enzyme function annotations agreed with our earlier EFICAz predictions (Figure 2). Third, by way of illustration, we identified a set of 64 previously hypothetical proteins whose biochemical functions have been recently characterized and found that in 96% of the cases, we correctly identified their three-field EC numbers, and in 84% of the cases, we could provide their fully detailed enzymatic activities (Tables 1, 2, 3). Achieving this level of precision is not trivial, considering that: (i) hypothetical proteins are the most difficult targets for automated function prediction [77], and (ii) the maximal sequence identity between the 64 hypothetical proteins and the EFICAz training enzymes has a median value of 25% (Figure 3). We were surprised to find a few cases among this set of 64 hypothetical proteins, where the annotation lag in databases was more than two years. It is difficult to estimate the full dimension of this problem; nevertheless, a systematic rescue of those annotations lost in the literature is very much needed, given the low number of experimentally verified functional assignments in the current databases [78].

There always exists a trade-off between precision and recall in the implementation of a predictive method. A consequence of our priorization of precision over recall is that the enzyme contents calculated based on our EFICAz predictions are lower-bound estimates (Figure 1D). However, EFICAz is still sensitive enough as to generate thousands of novel annotations. We believe that our novel predictions can be mined and correlated with other information sources to generate biologically significant hypotheses. As a proof of principle of this strategy, we have presented two examples, selected because of their potential impact on human health. Using the EFICAz based database on our website [32], we are confident that experts in different fields of biology will be able to discover many more such cases. To facilitate this task, the enzyme function assignments can be browsed on our website [32] according to species name, gene name, level of detail of the enzyme function prediction and EC number. Agreeing, disagreeing, or unique KEGG and EFICAz annotations, as well as EFICAz assignments for hypothetical proteins can also be selected and retrieved.

The main drawback of our analysis is the fact that we can only predict biochemical functions that are represented in our set of training enzymes by at least one sequence. Because of this requirement, the prediction of orphan enzymes is beyond the capabilities of both our approach and of all the current computational approaches for enzyme function inference. Orphan enzymes are defined as enzymatic activities that have been experimental measured, but not yet mapped to a gene product, i.e., EC numbers without known associated sequences [79,80]. According to a recent survey, the number of different orphan enzymes exceeds fifteen hundred, i.e. more than 39% of the known enzymatic activities [81].

We plan to periodically repeat the reannotation of all available genomes using updated versions of EFICAz and maintain all the annotations in a web-accessible database. By using the same version of EFICAz to reannotate all the available genomes simultaneously, rather than only the newly released genomes, we will keep the consistency of the annotations between genomes. This feature together with the full standardization of our annotations (EFICAz always reports EC numbers rather than enzyme names; although the latter are also provided) will be very useful for comparative genome analysis and automated metabolic pathway reconstruction, and will also facilitate the incorporation of EFICAz predictions to other functional databases.

## Methods

### EFICAz: Enzyme Function Inference by a Combined Approach

EFICAz is a combined approach designed specifically for high precision enzyme function inference [26]. It integrates the predictions of four independent methods: (i) *CHIEFc family based FDR recognition*: detection of Functionally Discriminating Residues (FDRs) in enzyme families obtained by a Conservation-controlled HMM Iterative procedure for Enzyme Family classification (CHIEFc), (ii) *CHIEFc family specific SIT evaluation*: pairwise sequence comparison using a CHIEFc family specific Sequence Identity Threshold (SIT), (iii) *High specificity multiple Prosite pattern recognition*: detection of multiple Prosite [82] patterns of high specificity, and (iv) *Multiple Pfam family based FDR recognition*: detection of FDRs in Multiple Pfam [83] enzyme families. In EFICAz, an enzyme family is defined as a group of proteins that are evolutionarily related and share the full four or the first three fields of their EC numbers. Each of the four methods is highly precise and able to generate unique assignments that are not detected by the other three components. Therefore, EFICAz makes an inference when one or more of the four component methods predict a particular enzyme function. The primary goal of EFICAz is predicting four-field EC numbers; however, when the highest level of detail for the enzyme function description cannot be confidently determined, EFICAz can provide three-field EC numbers. EFICAz and its components are fully described in our previous article [26].

### Training of different EFICAz versions

The source of annotated protein sequences for EFICAz is the UniProt Knowledgebase database (or UniProt for short) [42]. The UniProtKB/Swiss-Prot (or Swiss-Prot for short) component of UniProt is the source of the training enzyme sequences, that we require to be fully annotated with four-field EC numbers. A combination of the Swiss-Prot and the TrEMBL components of UniProt provides the source of sequences to prepare the heterofunctional multiple sequence alignments that are required for FDR selection by the Evolutionary Footprinting method [26]. For training of the *Multiple Pfam family based FDR recognition *component of EFICAz, we use the Pfam database [83].

We prepared three versions of EFICAz (2.0, 3.0 and 4.0) to benchmark the performance of our enzyme function prediction method in a situation mimicking periodic updates, and one version (5.0) to carry out enzyme function prediction on a multi-genome scale. The only differences among the various EFICAz versions are the releases of the different databases used for the training process. The sources of annotated protein sequences for versions 2.0, 3.0, 4.0 and 5.0 of EFICAz are the Releases 2.0, 3.0, 4.0 and 5.0 of UniProt, respectively. Table 5 shows the relevant statistics of the sequence data sources for the different versions of EFICAz. For training of the *Multiple Pfam family based FDR recognition *component of EFICAz versions 2.0, 3.0 and 4.0, we use the following Pfam database Releases: 15.0 of August, 2004 (based on UniProt 2.0), 16.0 of October, 2004 (based on UniProt 3.0) and 17.0 of March, 2005 (based on UniProt 4.0), respectively. For EFICAz version 5.0 we also use the Release 17.0 of Pfam. A detailed description of EFICAz training procedures can be found in our previous work [26]. See additional file 2: EFICAz_v5_enzymes.xls for a list of 2,061 enzyme types with four-field EC numbers and 203 enzymes types with three-field EC numbers recognized by EFICAz version 5.0.

### Benchmarking of EFICAz using annotated Swiss-Prot sequences

The results of the jackknife test presented in our previous work [26], showed that the original version of EFICAz generates highly precise enzyme function predictions. To corroborate that the precision of newer versions of EFICAz is comparable to that of the rigorously tested original version, we performed a benchmark in a scenario of periodic updates. Briefly, we select all the newly added Swiss-Prot sequences in the Release 5.0 of UniProt, i.e. not included in a given previous release of this database, and compare their functional annotations in UniProt 5.0 with our functional predictions using a version of EFICAz trained with the given previous release of UniProt. We tested the new sequences added to UniProt 5.0 since the release of UniProt 2.0 (33,475 sequences), UniProt 3.0 (18,325 sequences) and UniProt 4.0 (10,495 sequences), using the versions 2.0, 3.0 and 4.0 of EFICAz, respectively.

For a given enzyme function described by a four-field EC number, we calculate: precision = (true positives)/(true positives + false positives), and recall = (true positives)/(true positives + false negatives), where (i) true positives is the number of new sequences predicted by EFICAz as having the given enzyme function and annotated in UniProt 5.0 with that same function, (ii) false positives is the number of new sequences predicted by EFICAz as having the given enzyme function, but annotated in UniProt 5.0 with a different function, and (iii) false negatives is the number of new sequences annotated in UniProt 5.0 with the given function, but predicted by EFICAz as having a different enzymatic function or no enzymatic function at all. The enzyme sequences in UniProt are not evenly distributed over the different EC classes, i.e., some enzyme functions might be overrepresented. To reduce the bias towards the most populated enzyme functions, we first evaluate precision and recall for each individual enzyme type, and then average them across all types. On the other hand, because some newly added sequences are very similar to training enzymes (e.g., more than 90% sequence identity), they are much easier to predict than others. To reduce this second source of bias, we evaluate the performance of EFICAz according to different levels of maximal sequence identity of the test sequences to the training enzymes. Thus, for each enzyme type, we first select the test sequences whose sequence identities to any member of their corresponding training sets are not higher than a given value. Then, based on the selected testing sequences, we calculate the precision and recall of EFICAz for each of those enzyme types. Finally, for each version of EFICAz, we report the average precision and recall at different levels of maximal testing to training sequence identity.

Figure 4 shows the average precision (Fig. 4A–C), average recall (Fig. 4D–F) and number of predicted enzyme types (Fig. 4G–I), when EFICAz versions 2.0, 3.0 and 4.0 are applied to the sequences in UniProt 5.0 that were added since the release of UniProt 2.0, 3.0 and 4.0, respectively. Besides the results corresponding to all the observed enzyme types (blue curves in Figure 4), we also show those corresponding to enzyme types for which 10 or more training sequences were available (red curves in Figure 4). The average precision of any version of EFICAz is never below 94% (with standard deviations that never exceed 20%), irrespective of whether all enzyme types or only those with at least 10 or more training sequences are considered, and regardless of the sequence identity interval analyzed (Fig. 4A–C).

The average recall of EFICAz depends of the specific maximal testing to training sequence identity interval. Thus, when all enzyme types are considered, the recall ranges from 95% to 97% if no testing to training sequence identity restrictions are applied (100% sequence identity interval), but decreases to 69–74% at 40% sequence identity (Fig. 4D–F, blue curves). When only enzyme types with 10 or more training sequences are considered, the recall significantly improves, e.g. it ranges from 82% to 85% at 40% sequence identity (Fig. 4D–F, red curves). All the shown results correspond to four-field EC number predictions; the three-field EC number predictions follow the same trends, with slightly higher precision and recall (not shown). In general, these benchmark results clearly show that updated versions of our enzyme function inference method are very likely to perform as well as the original version of EFICAz.

### Genome sequence dataset

Using EFICAz version 5.0, we analyzed the protein sequences of all the genomes available in the *Genes *database Release 33.0+/03–05 (of March 5, 2005), a component of KEGG. The dataset comprises 866,142 coding sequences corresponding to 245 genomes. The whole dataset was processed in approximately 19.5 days, using 50 of the 1,000 nodes in our IBM e1350 cluster, powered by two 2.0 GHz dual core AMD Opteron 270 processors per node, i.e. the average running time of EFICAz in a single 2.0 GHz core was 3.24 minutes per genomic sequence. With the purpose of comparison, we collected the enzyme function annotation available for these sequences in the *Genes *database. We extracted the EC numbers (described at least at the level of the first three-fields) from the DEFINITION line in the corresponding gene entries of the Release 33.0+/03–05 and the Release 37.0+/03–07 (of March 7, 2006) of *Genes*. Table 1 includes the scientific names and taxonomic classification of all the organisms analyzed in this study.

### Search of hypothetical proteins annotated by EFICAz and recently characterized by experiments

To estimate the validity of our novel predictions, we first collected all the protein products predicted to be enzymes by EFICAz version 5.0 and defined as hypothetical or unknown in the Release 33.0+/03–05 of the *Genes *database (14,177 coding sequences). Predicting the function of unannotated proteins with high sequence similarity to enzymes that we used to train EFICAz can be considered a trivial exercise; e.g. EFICAz training enzymes may include homologs with very high sequence identity to a given protein labeled as hypothetical in databases due to the annotation lag problem [46]. Therefore, to make our test more demanding, we excluded from our list 254 hypothetical or unknown proteins exhibiting more than 60% sequence identity to any enzyme in the EFICAz version 5.0 training set. Then, for the remaining 13,921 proteins, we searched the PubMed database of May 26, 2006 [65] using their corresponding gene entry ids and names as a set of synonym query terms, resulting in 544 sequences linked to at least one article published in the last five years. To carry out the PubMed search, we used the Entrez Programming Utilities (eUtils) [84] from the National Center for Biotechnology Information (NCBI). After manual inspection to eliminate irrelevant abstracts, we obtained a set of 64 proteins whose biochemical functions have been experimentally determined and described with at least three-field EC numbers. Finally, we compared the experimentally-derived annotations of the 64 proteins with their EFICAz-predicted enzyme functions.

## Authors' contributions

AKA and WT participated in the design of the study, performed the enzyme function predictions and analyzed the results. AKA carried out the data mining work and drafted the manuscript. WT updated EFICAz, performed the benchmark, designed the website and helped to draft the manuscript. JS conceived of the study, participated in its design and coordination, and helped to draft the manuscript. All authors read and approved the final manuscript.

## Supplementary Material

Additional file 1 **Enzyme content**. Excel spreadsheet containing number of proteins, number of predicted enzymes and fraction of enzymes for the 245 species analyzed in this study. Click here for file

Additional file 2 **Enzymes types recognized by EFICAz**. Excel spreadsheet listing the 2,061 and the 203 enzyme types recognized by version 5.0 of EFICAz, described at the level of four-field or first three-field EC number, respectively. Click here for file

## Figures and Tables

**Table 1:** Species and taxonomic groups represented in the genome sequence dataset. The taxonomic information is from the NCBI Taxonomy database [85] and the three-letter code for the organisms is from KEGG [24]

**ARCHAEA**
**Euryarchaeota**: mja mmp mac mma mth mka afu hal hma tac tvo pto pho pab pfu tko
**Crenarchaeota**: ape sso sto pai
**Nanoarchaeota**: neq
**BACTERIA**

**Proteobacteria, Alphaproteobacteria**: rpr rty rco wol wbm ama eru erw erg mlo sme atu atc bme bms bja rpa bhe bqu ccr sil zmo gox
**Proteobacteria, Betaproteobacteria**: nme nma ngo cvi rso bma bps bpe bpa bbr neu eba
**Proteobacteria, Gammaproteobacteria**: eco ecj ece ecs ecc sty stt spt stm ype ypk ypm yps sfl sfx eca plu buc bas bab wbr bfl hin hdu pmu msu xfa xft xcc xac xoo vch vvu vvy vpa vfi ppr pae ppu pst aci son ilo cbu lpn lpf lpp mca ftu
**Proteobacteria, delta/epsilon subdivision**: hpy hpj hhe wsu cje cjr gsu dvu bba dps
**Firmicutes, Bacilli**: bsu bha ban bar baa bat bce bca bcz btk bli bld bcl oih gka sau sav sam sar sas sac sep ser lmo lmf lin lla spy spm spg sps spa spn spr sag san smu stc stl lpl ljo lac efa
**Firmicutes, Clostridia**: cac cpe ctc tte
**Firmicutes, Mollicutes**: mge mpn mpu mpe mga mmy mmo mhy uur poy mfl
**Firmicutes, Actinobacteria**: mtu mtc mbo mle mpa cgl cef nfa cdi sco sma twh tws lxx pac blo sth
**Fusobacteria**: fnu
**Planctomycetes**: rba
**Chlamydiae/Verrucomicrobia group**: ctr cmu cpn cpa cpj cpt cca pcu
**Spirochaetes**: bbu bga tpa tde lil lic
**Bacteroidetes/Chlorobi group**: cte bth bfr pgi
**Cyanobacteria**: syn syw syc tel gvi ana pma pmm pmt
**Chloroflexi**: det
**Deinococcus-Thermus**: dra tth ttj
**Aquificae**: aae
**Thermotogae**: tma
**EUKARYA**

**Fungi/Metazoa group**: hsa^1 ^mmu^1 ^rno^1 ^dre^1 ^dme cel
**Viridiplantae**: ath osa^1^
**Rhodophyta**: cme
**Fungi/Metazoa group**: sce ago cal spo ecu
**Mycetozoa**: ddi^1^
**Alveolata**: pfa cpv^1 ^cho^1^
**Euglenozoa**: tbr^1 ^lma^1^

^1^Incomplete genome project. aae: *Aquifex aeolicus*, aci: *Acinetobacter *sp. ADP1, afu: *Archaeoglobus fulgidus*, ago: *Ashbya gossypii*, ama: *Anaplasma marginale*, ana: *Anabaena *sp. PCC7120, ape: *Aeropyrum pernix*, atc: *Agrobacterium tumefaciens *C58 (Cereon), ath: *Arabidopsis thaliana*, atu: *Agrobacterium tumefaciens *C58 (UWash/Dupont), baa: *Bacillus anthracis *A2012, bab: *Buchnera aphidicola *Bp, ban: *Bacillus anthracis *Ames, bar: *Bacillus anthracis *Ames 0581, bas: *Buchnera aphidicola *Sg, bat: *Bacillus anthracis *Sterne, bba: *Bdellovibrio bacteriovorus*, bbr: *Bordetella bronchiseptica*, bbu: *Borrelia burgdorferi*, bca: *Bacillus cereus *ATCC 10987, bce: *Bacillus cereus *ATCC 14579, bcl: *Bacillus clausii*, bcz: *Bacillus cereus *ZK, bfl: *Blochmannia floridanus*, bfr: *Bacteroides fragilis*, bga: *Borrelia garinii*, bha: *Bacillus halodurans*, bhe: *Bartonella henselae*, bja: *Bradyrhizobium japonicum*, bld: *Bacillus licheniformis *DSM13, bli: *Bacillus licheniformis *ATCC 14580, blo: *Bifidobacterium longum*, bma: *Burkholderia mallei*, bme: *Brucella melitensis*, bms: *Brucella suis*, bpa: *Bordetella parapertussis*, bpe: *Bordetella pertussis*, bps: *Burkholderia pseudomallei*, bqu: *Bartonella quintana*, bsu: *Bacillus subtilis*, bth: *Bacteroides thetaiotaomicron*, btk: *Bacillus thuringiensis*, buc: *Buchnera aphidicola *APS, cac: *Clostridium acetobutylicum*, cal: *Candida albicans*, cbu: *Coxiella burnetii*, cca: *Chlamydophila caviae*, ccr: *Caulobacter crescentus*, cdi: *Corynebacterium diphtheriae*, cef: *Corynebacterium efficiens*, cel: *Caenorhabditis elegans*, cgl: *Corynebacterium glutamicum*, cho: *Cryptosporidium hominis*, cje: *Campylobacter jejuni *NCTC11168, cjr: *Campylobacter jejuni *RM1221, cme: *Cyanidioschyzon merolae*, cmu: *Chlamydia mu ridarum*, cpa: *Chlamydophila pneumoniae *AR39, cpe: *Clostridium perfringens*, cpj: *Chlamydophila pneumoniae *J138, cpn: *Chlamydophila pneumoniae *CWL029, cpt: *Chlamydophila pneumoniae *TW183, cpv: *Cryptosporidium parvum*, ctc: *Clostridium tetani *E88, cte: *Chlorobium tepidum*, ctr: *Chlamydia trachomatis*, cvi: *Chromobacterium violaceum*, ddi: *Dictyostelium discoideum*, det: *Dehalococcoides ethenogenes*, dme: *Drosophila melanogaster*, dps: *Desulfotalea psychrophila*, dra: *Deinococcus radiodurans*, dre: *Danio rerio*, dvu: *Desulfovibrio vulgaris *Hildenborough, eba: *Azoarcus *sp. EbN1, eca: *Erwinia carotovora*, ecc: *Escherichia coli *CFT073, ece: *Escherichia coli *O157 EDL933, ecj: *Escherichia coli *K-12 W3110, eco: *Escherichia coli *K-12 MG1655, ecs: *Escherichia coli *O157 Sakai, ecu: *Encephalitozoon cuniculi*, efa: *Enterococcus faecalis*, erg: *Ehrlichia ruminantium *Gardel, eru: *Ehrlichia ruminantium *Welgevonden (South Africa), erw: *Ehrlichia ruminantium *Welgevonden (France), fnu: *Fusobacterium nucleatum*, ftu: *Francisella tularensis*, gka: *Geobacillus kaustophilus*, gox: *Gluconobacter oxydans*, gsu: *Geobacter sulfurreducens*, gvi: *Gloeobacter violaceus*, hal: *Halobacterium *sp. NRC-1, hdu: *Haemophilus ducreyi*, hhe: *Helicobacter hepaticus*, hin: *Haemophilus influenzae*, hma: *Haloarcula marismortui*, hpj: *Helicobacter pylori *J99, hpy: *Helicobacter pylori *26695, hsa: *Homo sapiens*, ilo: *Idiomarina loihiensis*, lac: *Lactobacillus acidophilus*, lic: *Leptospira interrogans *serovar Copenhageni, lil: *Leptospira interrogans *serovar lai, lin: *Listeria innocua*, ljo: *Lactobacillus johnsonii*, lla: *Lactococcus lactis*, lma: *Leishmania major*, lmf: *Listeria monocytogenes *F2365, lmo: *Listeria monocytogenes *EGD-e, lpf: *Legionella pneumophila *Lens, lpl: *Lactobacillus plantarum*, lpn: *Legionella pneumophila *Philadelphia 1, lpp: *Legionella pneumophila *Paris, lxx: *Leifsonia xyli *xyli CTCB07, mac: *Methanosarcina acetivorans*, mbo: *Mycobacterium bovis*, mca: *Methylococcus capsulatus*, mfl: *Mesoplasma florum*, mga: *Mycoplasma gallisepticum*, mge: *Mycoplasma genitalium*, mhy: *Mycoplasma hyopneumoniae*, mja: *Methanococcus jannaschii*, mka: *Methanopyrus kandleri*, mle: *Mycobacterium leprae*, mlo: *Mesorhizobium loti*, mma: *Methanosarcina mazei*, mmo: *Mycoplasma mobile*, mmp: *Methanococcus maripaludis*, mmu: *Mus musculus*, mmy: *Mycoplasma mycoides*, mpa: *Mycobacterium avium *paratuberculosis, mpe: *Mycoplasma penetrans*, mpn: *Mycoplasma pneumoniae*, mpu: *Mycoplasma pulmonis*, msu: *Mannheimia succiniciproducens*, mtc: *Mycobacterium tuberculosis *CDC1551, mth: *Methanobacterium thermoautotrophicum*, mtu: *Mycobacterium tuberculosis *H37Rv, neq: *Nanoarchaeum equitans*, neu: *Nitrosomonas europaea*, nfa: *Nocardia farcinica*, ngo: *Neisseria gonorrhoeae*, nma: *Neisseria meningitidis *Z2491 (serogroup A), nme: *Neisseria meningitidis *MC58 (serogroup B), oih: *Oceanobacillus iheyensis*, osa: *Oryza sativa*, pab: *Pyrococcus abyssi*, pac: *Propionibacterium acnes*, pae: *Pseudomonas aeruginosa*, pai: *Pyrobaculum aerophilum*, pcu: *Parachlamydia *sp. UWE25, pfa: *Plasmodium falciparum*, pfu: *Pyrococcus furiosus*, pgi: *Porphyromonas gingivalis*, pho: *Pyrococcus horikoshii*, plu: *Photorhabdus luminescens*, pma: *Prochlorococcus marinus *SS120, pmm: *Prochlorococcus marinus *MED4, pmt: *Prochlorococcus marinus *MIT9313, pmu: *Pasteurella multocida*, poy: *Phytoplasma *sp. onion yellows, ppr: *Photobacterium profundum*, ppu: *Pseudomonas putida*, pst: *Pseudomonas syringae*, pto: *Picrophilus torridus*, rba: *Rhodopirellula baltica*, rco: *Rickettsia conorii*, rno: *Rattus norvegicus*, rpa: *Rhodopseudomonas palustris *CGA009, rpr: *Rickettsia prowazekii*, rso: *Ralstonia solanacearum *GMI1000, rty: *Rickettsia typhi*, sac: *Staphylococcus aureus *COL, sag: *Streptococcus agalactiae *2603, sam: *Staphylococcus aureus *MW2, san: *Streptococcus agalactiae *NEM316, sar: *Staphylococcus aureus *MRSA252, sas: *Staphylococcus aureus *MSSA476, sau: *Staphylococcus aureus *N315, sav: *Staphylococcus aureus *Mu50, sce: *Saccharomyces cerevisiae*, sco: *Streptomyces coelicolor*, sep: *Staphylococcus epidermidis *ATCC 12228, ser: *Staphylococcus epidermidis *RP62A, sfl: *Shigella flexneri *301, sfx: *Shigella flexneri *2457T, sil: *Silicibacter pomeroyi*, sma: *Streptomyces avermitilis*, sme: *Sinorhizobium meliloti*, smu: *Streptococcus mutans*, son: *Shewanella oneidensis*, spa: *Streptococcus pyogenes *MGAS10394, spg: *Streptococcus pyogenes *MGAS315, spm: *Streptococcus pyogenes *MGAS8232, spn: *Streptococcus pneumoniae *TIGR4, spo: *Schizosaccharomyces pombe*, spr: *Streptococcus pneumoniae *R6, sps: *Streptococcus pyogenes *SSI-1, spt: *Salmonella enterica *serovar Paratyphi A, spy: *Streptococcus pyogenes *SF370, sso: *Sulfolobus solfataricus*, stc: *Streptococcus thermophilus *CNRZ1066, sth: *Symbiobacterium thermophilum*, stl: *Streptococcus thermophilus *LMG18311, stm: *Salmonella typhimurium *LT2, sto: *Sulfolobus tokodaii*, stt: *Salmonella enterica *serovar typhi Ty2, sty: *Salmonella typhi *CT18, syc: *Synechococcus *sp. PCC6301, syn: *Synechocystis *sp. PCC6803, syw: *Synechococcus *sp. WH8102, tac: *Thermoplasma acidophilum*, tbr: *Trypanosoma brucei*, tde: i, tel: Thermosynechococcus elongatus, tko: *Thermococcus kodakaraensis*, tma: *Thermotoga maritima*, tpa: *Treponema pallidum*, tte: *Thermoanaerobacter tengcongensis*, tth: *Thermus thermophilus *HB27, ttj: *Thermus thermophilus *HB8, tvo: *Thermoplasma volcanium*, twh: *Tropheryma whipplei *Twist, tws: *Tropheryma whipplei *TW08/27, uur: *Ureaplasma urealyticum*, vch: *Vibrio cholerae*, vfi: *Vibrio fischeri*, vpa: *Vibrio parahaemolyticus*, vvu: *Vibrio vulnificus *CMCP6, vvy: *Vibrio vulnificus *YJ016, wbm: *Wolbachia *endosymbiont strain TRS of Brugia malayi, wbr: *Wigglesworthia brevipalpis*, wol: *Wolbachia *wMel, wsu: *Wolinella succinogenes*, xac: *Xanthomonas axonopodis*, xcc: *Xanthomonas campestris*, xfa: *Xylella fastidiosa *9a5c, xft: *Xylella fastidiosa *Temecula1, xoo: *Xanthomonas oryzae*, ype: *Yersinia pestis *CO92, ypk: *Yersinia pestis *KIM, ypm: *Yersinia pestis *Mediaevails, yps: *Yersinia pseudotuberculosis*, zmo: *Zymomonas mobilis*.

**Table 2:** Four-field EC number validation of EFICAz-predicted enzyme functions for 25 previously hypothetical proteins

Domain	Org.^1^	Gene name^2^	PMID^3^	True EC number^4^	Predicted EC number^5^	EC field Agreement^6^
Eukarya	hsa	54995	16261191	2.3.1.41	2.3.1.41	4
Eukarya	hsa	84779	16638120	2.3.1.88	2.3.1.88	4
Bacteria	ana	alr3351	15695431	6.3.2.2	6.3.2.2	4
Archaea	ape	APE0768	14551194	5.3.1.9	5.3.1.9	4
Bacteria	eco	b0581	15211520	6.3.2.2	6.3.2.2	4
Bacteria	ecc	c0667	15211520	6.3.2.2	6.3.2.2	4
Bacteria	mle	ML1399	15500449	4.6.1.1	4.6.1.1	4
Bacteria	pae	PA1167	15136569	4.2.2.3	4.2.2.3	4
Eukarya	cel	R07B7.11	15676072	3.2.1.49/3.2.1.22	3.2.1.22	4
Bacteria	mtu	Rv1647	15500449	4.6.1.1	4.6.1.1	4
Bacteria	mtu	Rv1700	12906832	3.6.1.13	3.6.1.13	4
Bacteria	mtu	Rv1885c	15654876	5.4.99.5	5.4.99.5	4
Bacteria	mtu	Rv2747	15838030	2.3.1.1	2.3.1.1	4
Bacteria	sfx	S0496	15211520	6.3.2.2	6.3.2.2	4
Bacteria	sfl	SF0488	15211520	6.3.2.2	6.3.2.2	4
Bacteria	spt	SPA0821	15547259	2.5.1.17	2.5.1.17	4
Bacteria	spt	SPA2151	15211520	6.3.2.2	6.3.2.2	4
Bacteria	sty	STY2255	15547259	2.5.1.17	2.5.1.17	4
Bacteria	stt	t0824	15547259	2.5.1.17	2.5.1.17	4
Archaea	tac	Ta1434	15044458	2.5.1.17	2.5.1.17	4
Bacteria	ece	Z0720	15211520	6.3.2.2	6.3.2.2	4
Bacteria	ecc	c0735	16411753	3.2.2.8	3.2.2.1	3
Archaea	mja	MJ0044	16621811	2.7.4.-	2.7.2.8	2
Archaea	mja	MJ0936	15128743	3.1.4.-	3.6.1.10	1
Bacteria	mtu	Rv0805	16313172	3.1.4.17	3.6.1.10	1

^1 ^The species names corresponding to the KEGG three letter codes are listed in the footnote of Table 1.

^2 ^Gene name from the *Genes *database of KEGG.

^3 ^PMID: PubMed Unique Identifier, the journal citation accession number for the most relevant record in PubMed supporting the experimentally-derived annotation [65].

^4 ^Experimentally-derived EC numbers.

^5 ^EFICAz-predicted EC numbers.

^6 ^Number of matching first n fields of the experimentally-derived and EFICAz-predicted EC numbers, with n = 1 to 4.

**Table 3:** Three-field EC number validation of enzyme functions predicted by EFICAz with four-field EC numbers for 12 previously hypothetical proteins

Domain	Org.^1^	Gene name^2^	PMID^3^	True EC number^4^	Predicted EC number^5^	EC field Agreement^6^
Bacteria	ecc	c2186	16077126	1.1.1.-	1.1.1.2	≤ 3
Bacteria	ecc	c5454	15489502	3.1.3.-	3.1.3.48	≤ 3
Bacteria	lla	L124252	15901700	2.1.1.-	2.1.1.14	≤ 3
Bacteria	pae	PA1032	16461666	3.5.1.-	3.5.1.11	≤ 3
Bacteria	sfx	S0029	11027694	3.2.2.-	3.2.2.1	≤ 3
Bacteria	sfl	SF0027	11027694	3.2.2.-	3.2.2.1	≤ 3
Bacteria	spt	SPA2330	15157072	2.7.1.-	2.7.1.2	≤ 3
Bacteria	spt	SPA4373	15489502	3.1.3.-	3.1.3.48	≤ 3
Bacteria	sty	STY0426	15157072	2.7.1.-	2.7.1.2	≤ 3
Bacteria	stt	t2471	15157072	2.7.1.-	2.7.1.2	≤ 3
Bacteria	ece	Z0035	11027694	3.2.2.-	3.2.2.1	≤ 3
Bacteria	ece	Z0493	15157072	2.7.1.-	2.7.1.2	≤ 3

^1 ^The species names corresponding to the KEGG three letter codes are listed in the footnote of Table 1.

^2 ^Gene name from the *Genes *database of KEGG.

^3 ^PMID: PubMed Unique Identifier, the journal citation accession number for the most relevant record in PubMed supporting the experimentally-derived annotation [65].

^4 ^Experimentally-derived EC numbers.

^5 ^EFICAz-predicted EC numbers.

^6 ^Number of matching first n fields of the experimentally-derived and EFICAz-predicted EC numbers, with n = 1 to 4.

**Table 4:** Three-field EC number validation of enzyme functions predicted by EFICAz with three-field EC numbers for 27 previously hypothetical proteins

Domain	Org.^1^	Gene name^2^	PMID^3^	True EC number^4^	Predicted EC number^5^	EC field Agreement^6^
Archaea	afu	AF1938	11790732	6.2.1.1	6.2.1.-	3
Bacteria	bsu	BG11467	14635137	2.3.1.-	2.3.1.-	3
Bacteria	bsu	BG11761	16242712	1.1.1.-	1.1.1.-	3
Bacteria	bth	BT4131	15952775	3.1.3.-	3.1.3.-	3
Bacteria	ecc	c1394	15157072	2.7.1.-	2.7.1.-	3
Bacteria	ecc	c2089	16253988	2.8.3.-	2.8.3.-	3
Bacteria	eco	b2873	11092864	3.5.2.-	3.5.2.-	3
Bacteria	cef	CE0356	15225990	2.3.1.-	2.3.1.-	3
Bacteria	lpf	lpl2377	16390437	2.7.3.-	2.7.3.-	3
Bacteria	lpp	lpp2524	16390437	2.7.3.-	2.7.3.-	3
Bacteria	lpp	lpp2599	11053398	2.1.1.-	2.1.1.-	3
Archaea	mja	MJ0883	15165845	2.1.1.31	2.1.1.-	3
Archaea	pho	PH1035	15737605	2.4.1.-	2.4.1.-	3
Archaea	pho	PH1915	16260766	2.1.1.-	2.1.1.-	3
Archaea	pho	PH1948	16245322	2.1.1.-	2.1.1.-	3
Bacteria	rpr	RP028	16364512	2.1.1.43	2.1.1.-	3
Bacteria	mtu	Rv0891c	15500449	4.6.1.1	4.6.1.-	3
Bacteria	mtu	Rv1500	16257960	2.4.1.-	2.4.1.-	3
Bacteria	mtu	Rv3225c	12715873	2.7.1.-	2.7.1.-	3
Bacteria	sco	SCO2599	12951512	3.1.4.-	3.1.4.-	3
Bacteria	spn	SP1051	12571357	2.7.1.-	2.7.1.-	3
Archaea	sto	ST0071	15212797	3.1.1.-	3.1.1.-	3
Archaea	sto	ST0723	16618099	1.5.1.30	1.5.1.-	3
Bacteria	ttj	TTHA1280	16511182	2.1.1.-	2.1.1.-	3
Bacteria	ypk	y0368	12923112	2.3.1.-	2.3.1.-	3
Bacteria	ype	YPO3632	16452420	2.3.1.-	2.3.1.-	3
Archaea	tac	Ta1419	14551194	5.3.1.8 5.3.1.9	6.1.1.-	0

^1 ^The species names corresponding to the KEGG three letter codes are listed in the footnote of Table 1.

^2 ^Gene name from the *Genes *database of KEGG.

^3 ^PMID: PubMed Unique Identifier, the journal citation accession number for the most relevant record in PubMed supporting the experimentally-derived annotation [65].

^4 ^Experimentally-derived EC numbers.

^5 ^EFICAz-predicted EC numbers.

^6 ^Number of matching first n fields of the experimentally-derived and EFICAz-predicted EC numbers, with n = 1 to 4.

**Table 5:** Source of Sequence Data for EFICAz training. The fifth column shows the number of enzymes annotated in Swiss-Prot with four-field EC numbers, which constitute the primary source for the training of EFICAz.

EFICAz version/UniProt Release	UniProt Release Date	Number of Sequences in UniProt	Number of Sequences in Swiss-Prot	Number of Enzymes in Swiss-Prot
2.0	Jul. 5, 2004	1,487,788	153,871	44,508
3.0	Oct. 25, 2004	1,612,609	163,235	47,144
4.0	Feb. 1, 2005	1,757,967	168,297	48,788
5.0	May 10, 2005	1,896,046	181,571	53,314
